# The impact of the harmonised education tariff on undergraduate general practice education in England: a qualitative interview study

**DOI:** 10.1186/s12909-026-09679-6

**Published:** 2026-06-17

**Authors:** Catharina Savelkoul, Sophie Park

**Affiliations:** Nuffield Department of Primary Care Health Sciences, University of Oxford, Radcliffe Primary Care Building, Oxford, OX2 6GG UK

**Keywords:** Medical education, Undergraduate, General practice education, Primary health care, Healthcare financing, Educational governance, Qualitative research

## Abstract

**Background:**

The harmonised undergraduate medical tariff, introduced in 2022, is a national (England) funding policy that provides consistent reimbursement for clinical teaching across all healthcare settings. For the first time, GP education is funded on a par with secondary care. There is no published evidence on its impact. This study examined how the tariff has affected the organisation, delivery, and sustainability of undergraduate GP education in England.

**Methods:**

Semi-structured interviews were conducted with twelve Heads of Undergraduate GP Teaching (HUGPTs), each from a different UK medical school; the findings are based on the ten interviews with HUGPTs at English medical schools (10/38, 26%). Data were analysed using Braun and Clarke's reflexive thematic analysis.

**Results:**

Six themes were identified. Participants consistently reported that the harmonised tariff improved transparency and reallocated budgetary authority to HUGPTs, enabling curriculum expansion, growth of central GP teaching teams, and the development of new educational initiatives. Better remuneration was perceived to improve recruitment and retention of teaching practices and enhance teaching quality, while accountability frameworks, including the Annex C guidance and reporting tool, provided external legitimacy and supported consistent implementation across medical schools.

**Conclusion:**

The harmonised tariff has been associated with reported improvements in undergraduate GP education. As oversight responsibilities transfer from NHS England to the DHSC, maintaining transparent budget-holder arrangements and accountability frameworks will be important to sustaining these improvements.

**Supplementary Information:**

The online version contains supplementary material available at 10.1186/s12909-026-09679-6.

## Introduction

Funding for undergraduate medical education in general practice has historically lagged behind that for hospital education. The Service Increment for Teaching (SIFT) system, introduced in 1976 to fund clinical teaching in NHS hospitals, initially excluded general practices [1, 2]. SIFT was only extended to GP teaching in 1995 at a token rate of £12.50 per student per half-day [1, 2]. Under this legacy model, medical schools delivered around 15% of clinical curriculum hours in general practice but received only about 7% of clinical placement funding for primary care [3].

The funding gap reflected several historical factors. SIFT was originally designed for hospital-based teaching where infrastructure costs were readily identifiable, whereas GP teaching costs were less visible and harder to quantify [1, 2]. Additionally, the lower prestige historically accorded to general practice within medical education [4–6] may have contributed to acceptance of lower funding levels. The dispersed, small-scale nature of GP placements (typically one or two students per practice) also meant that individual practices had limited bargaining power compared to NHS Trusts hosting large numbers of students [3].

By the 2010 s, this arrangement was widely regarded as outdated and inequitable [1, 4, 7, 8]. The number of academic GP departments had halved since 2002 and the duration of GP placements per student was stagnating or falling [3]. This was concerning given the Department of Health’s target for 50% of graduates to enter general practice [7, 8]. Adequate undergraduate GP experience is known to influence career choice, so insufficient and underfunded placements threatened future GP recruitment [9–11]. There was an urgent need for a new funding model for undergraduate primary care education in England.

Healthcare education funding in the UK is managed separately by each nation, with Scotland, Wales, and Northern Ireland operating under different funding arrangements. This paper focuses on England, where the harmonised tariff was implemented. While the findings from this funding reform may have relevance for the devolved nations, direct comparison is beyond the scope of this study.

In 2019, a national costing exercise by the Department of Health’s Primary Care Education Working Group found GP teaching costs were comparable to secondary care, yet GP practices were reimbursed at barely half this level [1]. This led to extensive negotiations through the National Tariff Advisory Group, bringing together the Department of Health and Social Care in England (DHSC), Health Education England and other key stakeholders [8].

**Fig. 1:**
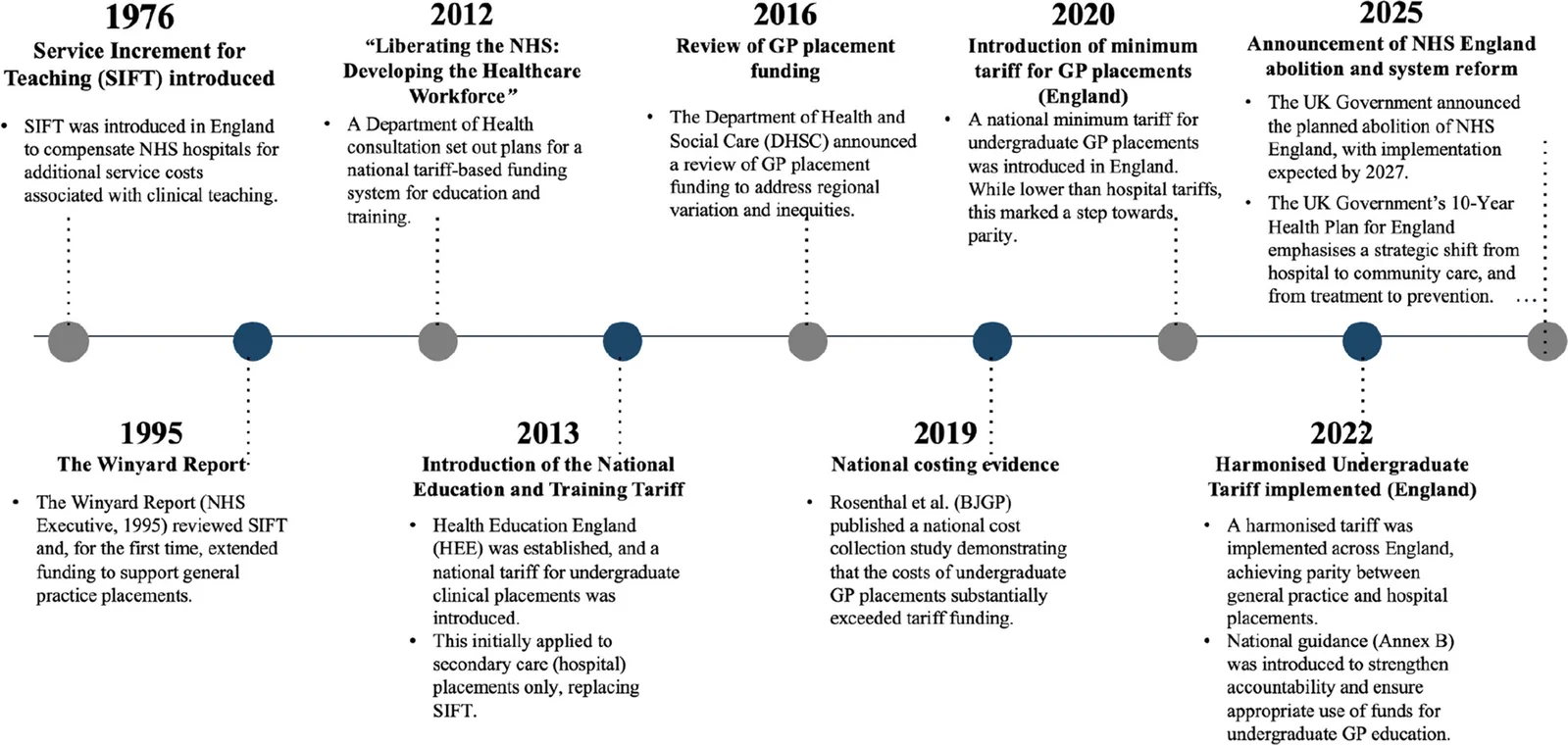
Key changes in funding and tariff arrangements for undergraduate general practice placements leading up to the harmonised undergraduate tariff, 1976–2025 (figure created by authors). SIFT = Service Increment for Teaching; HEE = Health Education England; DHSC = Department of Health and Social Care; BJGP = British Journal of General Practice; NHS = National Health Service. Policy developments apply to England unless otherwise stated

In March 2022, the DHSC introduced guidance for a new undergraduate tariff providing consistent national funding for clinical teaching across all settings, with GP teaching funded on a par with secondary care for the first time (Fig. 1) [8, 12]. The undergraduate medical education tariff is funding paid to placement providers to cover the costs of hosting and teaching medical students during clinical placements. For secondary care, NHS England pays NHS Trusts directly based on placement activity reported by medical schools. For primary care, given the distributed nature of GP teaching across multiple small settings, NHS England pays Higher Education Institutions (HEIs), which devolve the funding to their Head of Undergraduate GP Teaching (HUGPT). The HUGPT then allocates funds to teaching practices based on student numbers and placement activity. The policy introduced two organisational changes: (1) the HUGPT became the budget holder for all undergraduate primary care funding devolved by NHS England (NHSE); and (2) HEIs were asked to prepare an annual accountability report (piloted in 2022/2023) [13]. Annex C sets out the scope and conditions for the use of the undergraduate primary care tariff, specifying which activities and costs are eligible for funding, requiring the designation of a HUGPT as budget holder, and outlining the reporting requirements for HEIs [14].

The new undergraduate tariff was expected to improve undergraduate GP education by introducing a *‘bespoke funding model*’ ([8], p.257]) that recognises the unique organisational requirements of GP education. It also sought to formalise the role of HUGPTs by granting ‘*a level of agency commensurate with their responsibilities*’ ([8], p.258]) and tools to oversee spending decisions. However, there is no published empirical evidence on its impact. This study addresses this gap by examining how the harmonised tariff has impacted the opportunities and challenges of coordinating, delivering and sustaining undergraduate GP education in England.

This paper is both timely and necessary; in March 2025, the UK government announced that NHSE will be abolished, with its functions, including oversight of undergraduate primary care education, transferring to the DHSC within two years [15]. High-quality undergraduate GP education is also critical to delivering the UK Government's 10-Year Health Plan for England's mandate to shift care to the community and achieve the target of 50% of graduates entering general practice [7, 8, 16]. This paper therefore examines the successes and remaining challenges of the current funding model to inform its transition and future sustainability.

This study addressed three research questions: (1) How has the harmonised tariff affected the organisation and governance of undergraduate primary care education? (2) What impact has the tariff had on recruitment and retention of teaching practices and faculty? (3) How do HUGPTs perceive the effects on curriculum delivery and teaching quality?

## Method

### Study design

We conducted a qualitative study using semi-structured interviews with Heads of Undergraduate GP Teaching (HUGPTs) across UK medical schools [17]. Data were analysed using Braun and Clarke's reflexive thematic analysis (RTA), applying the 'practical' variant suited to applied health and education research [18, 19]. The study protocol and initial topic guide were pre-registered on the Open Science Framework prior to data collection (10.17605/OSF.IO/UAFDS).

### Setting and participants

Each UK medical school typically has one designated HUGPT who leads the central GP teaching team (CGPT) and coordinates undergraduate primary care education. HUGPTs are typically senior academic GPs who combine clinical practice with educational leadership roles; they may hold qualifications in medical education (e.g., PGCert, Masters, or PhD in medical/clinical education) and are usually members of the Society for Academic Primary Care (SAPC) National Heads of Teaching Committee. SAPC is the UK's professional body for academic primary care, and its National Heads of Teaching Committee includes HUGPTs from across UK medical schools. Terminology for this role and the academic 'home' of GP teaching teams vary: some are based in central medical school departments, but most sit within departments of primary care.

An invitation email with attached participant information sheet was circulated via the SAPC Heads of Teaching Committee email list in May 2025. Interested HUGPTs responded directly to the first author, who scheduled interviews. SAPC membership is not mandatory for HUGPTs, but the Heads of Teaching Committee provides a forum through which most HUGPTs can be reached. Twelve HUGPTs, each from a different medical school, were recruited: ten from English medical schools and two from devolved nations, included to provide perspective from settings operating under different funding arrangements. As the harmonised tariff applies only to England, the findings are based on the ten interviews with HUGPTs at English medical schools (10/38, 26%). The sample achieved diversity across institution type, geographical setting, curriculum model, and scale of GP teaching (ranging from 2 to 12 weeks per student), and participants differed in time in role and gender (see Table 1).

**Table 1:** Characteristics of participating medical schools: overview of the 12 UK medical schools included in the study

	**Medical school**	**Russell group**	**Medical School est**	**Cohort size**	**Course**	**Teaching style (overall)**
English Medical Schools	1	X	New	Medium	Problem-based	PBL-based
	2	X	New	Medium	Integrated	Mixed
	3	✓	New	Medium	Problem-based	PBL-based
	4	✓	Established	Medium	Integrated	Mixed
	5	✓	Established	Large	Integrated	Case-based
	6	✓	Established	Large	Integrated	Mixed methods
	7	✓	Established	Medium	Traditional	Traditional
	8	✓	Long-established	Large	Integrated	Mixed
	9	✓	Long-established	Large	Problem-based	PBL-based
	10	✓	Long-established	Large	Integrated	Mixed
Non-English UK medical school	11	✓	Long-established	Medium	Problem-based	PBL/CBL
	12	✓	Long-established	Medium	Integrated	Mixed

Abbreviations and definitions: Est. = Established (year medical school was founded): New = founded post-2000; Established = founded 1950–1999; Long-established = founded pre-1950. Cohort size: Medium = < 300 students per year; Large = > 300 students per year. Course types: Integrated = curriculum integrating basic science and clinical teaching throughout; Traditional = curriculum with distinct pre-clinical and clinical phases; Problem-based = curriculum structured around problem-based learning

Teaching style abbreviations: *GP* General Practice, *PBL* Problem-Based Learning, *CBL* Case-Based Learning, *TBL* Team-Based Learning Russell

Group: ✓ = member; ✗ = non-member. The Russell Group is a self-selected association of 24 research-intensive UK universities

### Data collection

Interviews were conducted between June and August 2025 via Microsoft Teams, each lasting 30–60 min. The topic guide (Table 2) was designed to reflect our research questions and was refined iteratively as preliminary analysis identified new areas warranting exploration. Verbal informed consent, including permission to record, was obtained at the start of each interview. Interviews were audio-recorded and transcribed verbatim using automated transcription software; transcripts were verified against the original recordings by the first author (CS). Data collected was stored securely on encrypted University servers in compliance with the institutional data governance requirements.

**Table 2:** Interview topic guide

Topic (prompts, if necessary)	
• Can you tell me a bit about your role as Head of GP Teaching? (How long have you been in post, what does your role involve?) • Can you tell me a bit about your course? (When and how do you offer teaching and placements, student numbers, assessment) • Have you been the tariff budget holder? (Implications, changes since introduction) • Can you talk me through the changes due to the harmonised tariff? (Curriculum footprint, teaching, GP placements, student numbers, assessment, challenges) • Do you feel tariff changes have benefitted you or others? (Students, patients, GP tutors, colleagues) • Do you think tariff changes have impacted student experience? (Attitudes towards GP, other aspects) • What works about the current model? (Changes wanted, worries) • Did you use the NHSE primary care tariff reporting card? (Usefulness, possible differences without it) • Have you referred to the Annex C document? (Helpfulness, dilemmas, impact without it) • Is there anything else you think we've missed or any questions you have?	

### Data analysis

CS coded the ten transcripts from HUGPTs at English medical schools inductively in NVivo 15 following Braun and Clarke's six phases of RTA [18, 19]: familiarisation, coding, generating initial themes, reviewing themes, defining and naming themes, and writing up. After coding six transcripts, CS and SP met to review the coding frame and refine codes. Following coding of all 10 transcripts, themes were developed through team discussion. Where interpretations differed, we returned to the original data and resolved disagreements through discussion until consensus was reached. The final thematic structure (Table 3) was agreed by both authors. The two transcripts from participants in devolved nations did not form part of the analytic dataset from which codes and themes were developed. They were read to inform the interpretation of findings in relation to the wider UK context. Saturation was assessed in relation to the ten interviews with HUGPTs at English medical schools, as the harmonised tariff applies only in England. No new themes were identified after the ninth interview.

**Table 3:** Thematic coding framework: major themes and subthemes identified in interviews with Heads of Undergraduate GP Teaching

Major theme	Subthemes	Working definition
1. Budget control, transparency and organisational change	Greater transparency and oversight	Improved visibility over how undergraduate primary care education funding is allocated and spent
	Gaining formal budget control	HUGPTs acquiring direct managerial authority over the undergraduate primary care education budget within their institution, including the ability to determine how funds are distributed
	Institutional cultural change	Shifts in institutional attitudes toward, and recognition of, the value of undergraduate GP education
2. Curriculum expansion and educational innovation	Expansion of GP teaching within the curriculum	Increases in the volume of GP teaching within the undergraduate medical curriculum, including extended placement blocks
	Central GP Teaching Team (CGPT) growth	Growth in the size, capacity, or scope of central GP teaching teams
	Development of educational initiatives	New teaching programmes or educational resources developed or introduced with support from the tariff
3. Placement capacity	Recruitment of new teaching practices	Ability to bring new GP practices into the undergraduate teaching network
	Retention of existing teaching practices	Ability to maintain the engagement and commitment of GP practices already involved in undergraduate teaching
	Ongoing concerns over capacity	Concerns about the future ability to meet rising placement demand, including the impact of increasing student numbers, new medical schools, and competing training requirements
4. Teaching quality and assurance	Tutor engagement	Efforts to support, develop, or sustain the engagement and educational skills of GP tutors involved in undergraduate teaching
	Quality assurance processes	Systems and processes for monitoring, evaluating, and improving the quality of GP placements and teaching
5. Impact on student experience	Perceptions of general practice	How the tariff and associated changes to GP teaching influenced students’ views of general practice as a potential career
	Practical benefits for students	Improvements in placement logistics attributed to tariff-funded changes
6. Accountability frameworks	Annex C as guidance and legitimacy tool	Use of Annex C to define the scope of permissible tariff expenditure and to negotiate with institutional finance teams
	Use of the NHSE reporting tool	Experiences of the mandatory annual reporting mechanism for tariff expenditure, including its role in demonstrating accountability to NHS England
7. Challenges and limitations of the tariff	Administrative burden of the reporting tool	Time, resource, and administrative costs associated with completing mandatory reporting requirements
	Difficulties interpreting Annex C in edge cases	Challenges in determining whether specific activities or costs fell within the scope of Annex C guidance

The number of participants contributing to each theme is reported in the Results narrative, where the denominator (10 English participants for tariff-specific findings; 12 for the full sample) can be specified in context

*Abbreviations*: *HUGPT* Head of Undergraduate GP Teaching, *CGPT* Central GP Teaching Team, *GP* General Practice, *NHSE* NHS England, *Annex C* Annex C of the 2022–23 Education and Training Tariff guidance document

The collaborative approach to analysis emphasised researcher subjectivity as a resource while using dialogue to enhance rigour [18, 19]. A draft of the analysis was circulated to participants prior to submission to verify that themes accurately reflected their perspectives. Supplementary Table S1 maps each theme and subtheme to the relevant topic guide questions.

Quotations were selected to illustrate the range and depth of views within each theme. For each subtheme, we reviewed all relevant coded excerpts and selected quotations that were: (a) representative of the majority view where consensus existed, (b) illustrative of the range of perspectives where views diverged, and (c) clear and articulate in expressing the point.

## Results

Six key themes were identified regarding the impact of the harmonised tariff: budget control and organisational change; curriculum change; placement capacity; teaching quality and assurance; impact on student experience; and the use of accountability frameworks. As the harmonised tariff applies only to England, the findings below draw on the ten interviews with HUGPTs at English medical schools. The two interviews with participants from devolved nations, conducted after saturation had been reached, confirmed that challenges in recruitment, retention, and funding for GP education are shared across the UK. These interviews are not drawn upon directly in the findings that follow.

### Budget control, transparency and organisational change

Before the tariff, most participants reported limited oversight and control over how much funding was devolved to primary care or how it was spent within their institution. Under the harmonised tariff, Annex C guidance formally designates the HUGPT as the budget holder for undergraduate primary care funding.

#### Greater transparency and oversight

Participants consistently reported that this change improved transparency in how undergraduate primary care funding is and should be allocated.

‘It is now much more transparent with the medical school about the money coming in being spent appropriately on primary care education.’- HUGPT 2

#### Gaining formal control of the budget

Most participants noted that being formally designated as the budget holder strengthened their ability to advocate for the needs of undergraduate primary care education within their medical school:

‘One of the outcomes [of the harmonised tariff] is more power and control to the HUGPT in the local financial arrangements … they can argue the case when they need more staff and more resources in a way that didn’t used to be possible.’ - HUGPT 7

Participants also reported that this new arrangement ensured the appropriate allocation and use of funds for GP education, as one explained:

‘Without the HUGPT’s sign off that the money's been spent appropriately … it shouldn't be released to other people.’- HUGPT 1

#### Institutional cultural change

There is substantial evidence of a persistent hidden curriculum [4–6] in UK medical schools, which portrays hospital specialties as more prestigious while denigrating general practice. Within this context, several participants observed that the funding parity challenged this prevailing cultural divide:

‘There's just such value to having a harmonised tariff … it goes back to that “Are you going to be just a GP or are you going to specialise?” … the harmonised tariff goes some way in making us feel that we are equivalent to secondary care.’ - HUGPT 7

The harmonisation of the tariff between primary and secondary care acted as a *legitimacy signal* [20], indicating that undergraduate education in general practice is just as important as hospital-based teaching:

‘To have a funding model where you have apparent parity between primary care and secondary care … is very clearly a very progressive step.’ - HUGPT 4

### Curriculum expansion and educational innovation

Before the harmonised tariff, undergraduate GP education had plateaued or, in some cases, declined, with the amount of teaching per student falling in several medical schools [3, 21]. Addressing this stagnation became a priority; the *By Choice—Not by Chance* [4] report called for better and more integration of GP education into curricula.

Most participants described how the formal designation as budget holder enabled them to achieve this curriculum expansion because the shift in resource control [21] reduced their dependence on the central medical school and allowed them to align funding more closely with the priorities for GP education.

#### Expansion of GP teaching within the curriculum

Most participants indicated that the harmonised tariff served as a catalyst for expanding GP teaching, addressing the ‘*lamentable structural inequality*’ ([22], p.164]) where the GP footprint in curricula was not matched by funding. For the first time, allocations were proportionate to the curriculum footprint, providing the leverage to plan, resource, and deliver expanded undergraduate GP education:

‘[The harmonised tariff] was really helpful because we were doubling up the amount of time students had in GP placement.’ - HUGPT 5

A small number of participants had not yet used the new tariff for curriculum change, although some described plans to do so:

‘The amount of clinical [GP] exposure is falling behind other institutions … there has been a lot of discussion about how we could use tariff money to facilitate that increase.’ - HUGPT 8

#### Central GP team (CGPT) growth

The scale, heterogeneity, and dispersed nature of UK primary care mean that, in most schools, key elements of undergraduate GP placements are delivered centrally by a medical school GP teaching team (CGPT). These include major placement management and coordination (often across 100 + practices), alongside financial, quality, and planning processes. Many schools also provide centrally delivered clinical teaching by GPs [4]. The tariff supports these activities [14]. Some participants reported that the additional funding provided through the new funding model facilitated recruitment to the CGPTs, with previously small and under-resourced teams reliant on ad hoc staff now able to grow:

‘We’ve historically been a very small team, underfunded in terms of administration support and clinical teachers, and so we were able to expand the team.’ - HUGPT 1

#### Development of educational initiatives

New models of delivery and innovative approaches are needed to prepare students for the changing healthcare system [4, 16]. Most participants reported that the harmonised tariff provided the means to develop and implement new primary care educational initiatives:

‘We couldn't be as creative as we can be now … we've got new initiatives such as the clinical humanities project or the simulated clinics.’ - HUGPT 6

### Placement capacity

Consistent with the aim of establishing a ‘*bespoke funding model*’ ([8] ,p.258]) for undergraduate GP education, the harmonised tariff enabled most participants to increase their payments to teaching practices, bringing reimbursement closer to the true costs of delivering placements [1]:

‘[The harmonised tariff] meant that I could pay my GP teachers that are delivering placement activity better.’ - HUGPT 3

#### Recruitment of new providers

Most participants reported that the harmonised tariff improved their ability to recruit new teaching practices, whereas previously they had struggled:

‘I have noticed that [practices] are now saying yes.’ - HUGPT 3

‘We were able to add in funding to encourage practices who were wavering to and also recruit a couple of new quite big practices.’ - HUGPT 1

This aligns with existing evidence that inadequate remuneration has long been a barrier to recruitment of teaching practices [23, 24].

#### Retention of existing providers

Participants consistently noted that, before the new funding model, many practices considered withdrawing as previous financial arrangements were no longer viable; the new tariff helped retain them:

‘We did get [practices] saying “we can't afford to do this anymore”. That hasn't happened once since the new tariff came in.’ - HUGPT 2

#### Ongoing concerns over capacity

A few participants raised concerns about future placement capacity, citing rising medical student intakes, new medical schools [25–28], and increased training demands from postgraduate GP trainees and allied health professionals; together these pressures risk creating competition:

‘Many new medical schools are going to be competing for the same placements and allied healthcare professionals … wanting to have placement activity in general practice.’ - HUGPT 3

At one medical school, the harmonised tariff was reported to have mitigated some of these concerns:

‘We were quite worried about another medical school to be in our area … that was why we've tried to increase our local practices … we're almost a bit too successful … if we did have an expansion of medical students … we'd probably be OK.’ - HUGPT 7

### Improving authentic teaching quality and assurance

#### Tutor engagement

Most participants perceived that change in remuneration improved tutor satisfaction and, in some cases, the quality of teaching:

‘[The tariff] made the GP tutors happier because … they're getting what they deserve.’ - HUGPT 6

‘[GP tutors] do a better job if they feel they're being rewarded appropriately for it.’ - HUGPT 2

#### Quality assurance

As designated budget holders, HUGPTs now have oversight of tariff allocation and can directly link funding decisions to quality assurance processes. Some participants described how this integration allows them to leverage change in a timely and effective way:

‘[The tariff] gives us more clout … to ensure quality… if a practice is giving lots of self-directed learning sessions, we can say, “Hang on. You're getting paid this much… you need to be giving them more teaching”.’ - HUGPT 7

### Impact on student experience

Teaching activity is always balanced, integrated and delivered alongside clinical care. The funding of teaching, therefore, has to ensure that both can flourish in synergistic ways. Some participants reported that students appeared to feel more valued by practices, as they were perceived to be generating appropriate income for the practice rather than being a burden.

#### Perceptions of general practice

A few participants suggested that improved remuneration and tutor engagement led to more positive student experiences and attitudes towards general practice:

‘[The harmonised tariff] has improved positivity towards general practice from our students.’ - HUGPT 5

However, some HUGPTs were careful not to overclaim on this point, until further evidence is available:

‘I think it would be quite tenuous to make a direct link … because there are so many factors.’ - HUGPT 4

#### Practical benefits for students

With more practices willing to teach following the tariff, some participants reported that their schools have been able to improve placement logistics for students:

‘I have been able to move [the placements] much more closely because local practices have now said we'll take students.’ - HUGPT 3

A few participants described using the harmonised tariff to act on student feedback:

‘We're providing the tariff funding now for a full day [in the GP surgery] because students were complaining that … half a day is not enough.’ - HUGPT 9

### Accountability frameworks

#### Annex C as guidance

Guidance is set out in Annex C of the DHSC education and training tariff guidance [14]. Most participants found this guidance document very helpful for clarifying appropriate tariff use for themselves and in negotiations with colleagues to define the boundaries of primary care funding. Many participants described using Annex C as a reference tool in discussions about the use of the primary care tariff, as it provides both *internal clarity* and *external legitimacy*.

‘[Annex C] has been useful because it means I can say yes and no to things.’ - HUGPT 3

#### Use of the reporting tool

Most participants viewed external oversight by a national body (currently NHSE, moving to DHSC) through the reporting tool as necessary to keep the operationalisation of the tariff fair and consistent across medical schools. Piloted by eight HUGPTs in 2022/2023, the tool was later rolled out nationally. Most have prepared it each year in case of an NHSE formal request.

Many participants emphasised the tool's importance and expressed that reporting should stay mandatory in future to ensure that budget allocation was compliant with the requirements of Annex C.

‘[The reporting tool] makes sure that medical schools are carrying out the right processes and that heads of GP teaching are involved.’ - HUGPT 6

Consistent with Annex C, some participants emphasised the importance of regular tariff meetings with medical schools to review expenditure and ensure alignment with national guidance.

## Challenges and limitations of the tariff

While participants' overall assessment of the harmonised tariff was positive, several challenges and limitations were identified. Some participants noted the administrative burden of completing the reporting tool, which some found time-consuming.

A few participants described challenges in interpreting the scope of Annex C guidance, particularly around edge cases where the boundaries of permissible expenditure were unclear.

## Discussion

### Summary

High-quality undergraduate GP education is critical to meeting the plans outlined in the 10-Year Health Plan [16] to shift care from hospitals to the community and to achieve the target for 50% of graduates to enter general practice [7, 8]. With NHSE set to be abolished and its functions transferred to the DHSC within two years [15], clear evidence of what has worked is crucial to support the appropriate continuation and strengthening of these funding arrangements in the future. This paper shows how this important change in funding model has been associated with reported improvements in the organisation, delivery and quality assurance of undergraduate primary care medical education across England. Future research using quantitative methods is needed to measure the magnitude of these changes and establish causal relationships. The following mechanisms may explain these reported improvements:

Designating HUGPTs as budget holders and reallocating budgetary decision-making authority to them increased transparency and repositioned the financial resources and decision-making power [21] with those directly responsible for delivering teaching. HUGPTs reported that this change reduced their dependence on the central medical school and enabled them to align funding more closely with the priorities for primary care education. It also reduced information asymmetry; while some HUGPTs already had oversight, the formal designation as budget holder ensured that all now have full visibility and accountability, directing allocated funds specifically to primary care education and reducing the risk of these funds being absorbed into other medical school budgets [29] (Table 4).

**Table 4:** Theoretical lenses applied to post-harmonised tariff changes in undergraduate GP education: mapping findings to PAT, RDT and Institutional theory

Theory	What it explains	Evidence
Principal-Agent Theory (PAT) (Jensen & Meckling, 1976; Ross, 1973)	How reducing information asymmetry and granting HUGPTs budget sign-off aligned spend with intended primary care educational uses; strengthened monitoring/QA	*Theme 1, subtheme 1*: mechanics of control and oversight; HUGPTs describing new budget oversight
Resource Dependence Theory (RDT) (Pfeffer & Salancik, 1978)	How access to and control of tariff funds shifted dependencies and leverage	*Theme 1, subtheme 2*:* Themes 2–3*—leverage; HUGPTs reporting ‘more influence, power and control’ with central finance
Institutional Theory (DiMaggio & Powell, 1983 [30]; Scott, 1995/2001 [31])	How tariff parity acted as a legitimacy signal, improving GP education’s status (challenging the hidden curriculum)	*Theme 1, subtheme 3*: cultural/legitimacy shift; HUGPTs noting ‘the narrative matches the pay.’

In turn, this reallocation [21] and greater transparency [29], enabled most HUGPTs to expand and better integrate GP education in the curriculum, grow the central GP team (CGPT) and introduce new educational initiatives. In six English medical schools, this also allowed for increased payments to teaching practices. Participants reported that better remuneration improved recruitment and retention of placement providers, which was perceived to increase the quantity and quality of clinical teaching. As Barber et al. [24] showed, practices that feel recognised and supported integrate teaching into the fabric of care; where support is thin, teaching drifts to the margins. The harmonised tariff matters, then, not only as a funding reform but as a relational one. Further research is needed to understand whether and how changes in remuneration influence teaching quality and student career intention.

**Fig. 2:**
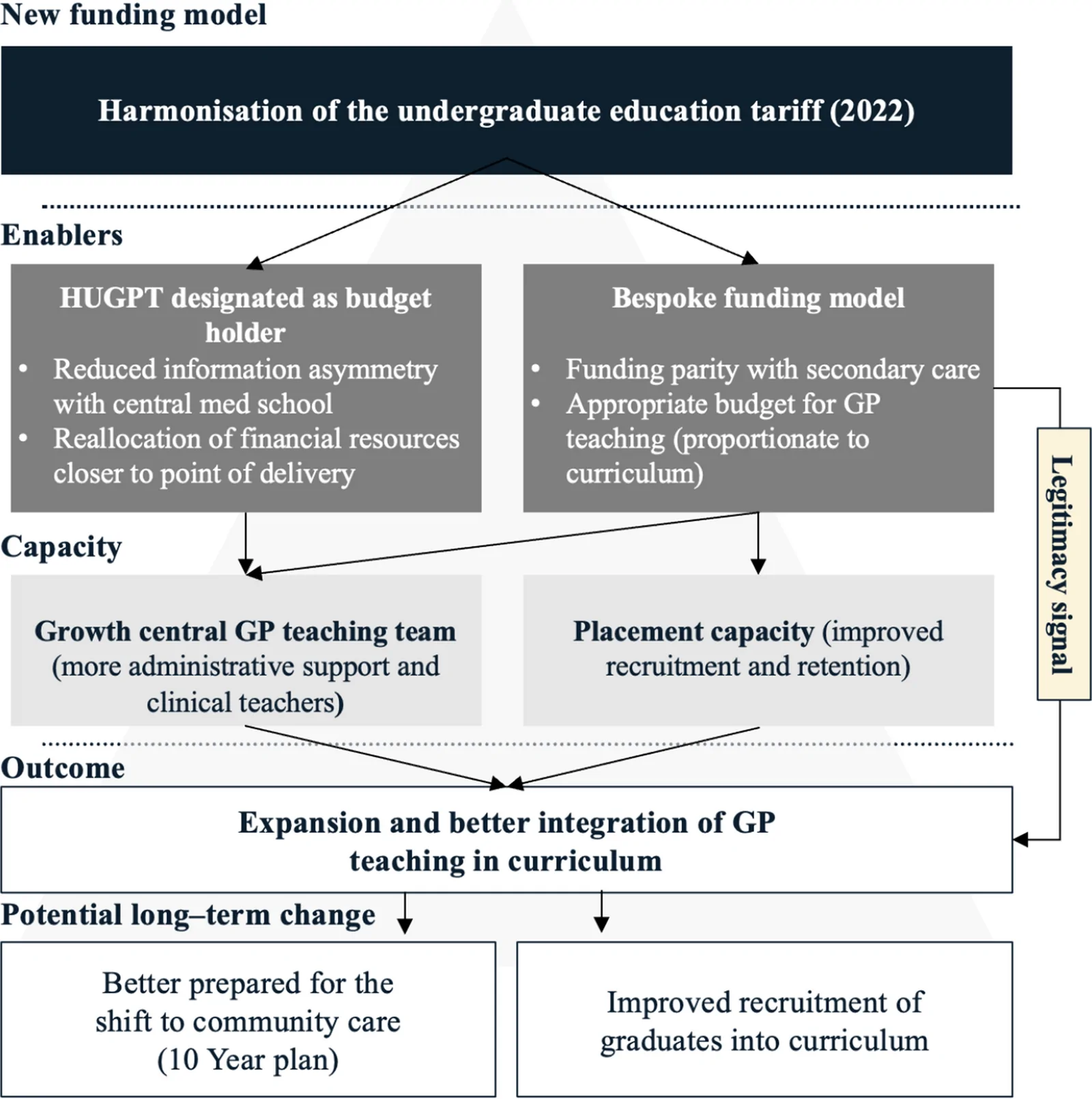
Shows the mechanisms by which the harmonised undergraduate education tariff improved the coordination and delivery of undergraduate GP teaching (figure created by authors)

The mechanisms described above appear to work synergistically. These include increased transparency, HUGPT budget control, curriculum expansion, improved practice remuneration, and enhanced recruitment and retention. Participants described how each element reinforced the others: for example, better remuneration improved recruitment, which increased placement capacity, which enabled curriculum expansion. When taken together, these interconnected changes represent a meaningful improvement in the delivery of undergraduate GP education, though further quantitative research is needed to establish the magnitude and causality of these effects (see Fig. 2).

Previously, there were concerns that the urgent recommendations from the *Wass* report [4] (e.g. equitable funding for GP placements and fuller curricular integration) were becoming ‘*static articles of faith*’ ([22], p.164]), but our findings indicate progress. The harmonised tariff represents a significant step in countering the legacy of the (Flexner-influenced) model of medical education [32], which prioritised hospital-based, subspecialty-focused training and inadvertently devalued generalism and community-based care [33], while simultaneously challenging the persistent hidden curriculum [4, 5, 20]. The harmonisation of the tariff functioned as a *legitimacy signal* that reframes undergraduate GP education as core rather than peripheral within medical schools [30, 31].

Looking ahead, concerns were raised about future placement capacity with the increase in medical student intakes, the opening of new medical schools [25–28] and the growing numbers of postgraduate GP trainees and allied healthcare professionals. The national HUGPT group could play an important coordinating role by helping to balance payment rates and adapt placement expectations to local curriculum and contextual needs.

### Strengths and limitations

The sample of 10 HUGPTs represented approximately one-quarter (26%) of English medical schools. Thematic saturation was achieved by the ninth interview, suggesting the sample was sufficient for the study aims. As designated budget holders, HUGPTs are a key stakeholder group and the most directly placed to describe changes following the tariff and, therefore, an important first group to research. However, as the beneficiaries of greater budgetary control under the tariff, HUGPTs may have been predisposed to view the changes favourably. The data collection period of three months was determined by the academic timeline of the doctoral research project, which may have limited the potential sample size; however, the achievement of thematic saturation and the representation of approximately one-quarter of English medical schools suggests the sample was adequate for the study aims. Further research should explore other perspectives about this topic, including from medical school finance teams and teaching practice staff, and, in due course, quantitative data to assess impacts on placement capacity, faculty development, curriculum contribution, and graduate career outcomes.

While this study focused on England where the harmonised tariff applies, the two comparative cases from devolved UK nations suggest that the challenges of funding, recruiting, and retaining teaching practices are shared across the UK. Lessons from the English experience may therefore have relevance for policy development in Scotland, Wales, and Northern Ireland, though direct transferability would require consideration of local funding arrangements and governance structures.

### Implications for policy

This analysis provides important insights into how changes in funding models impact the coordination, practice and delivery of clinical education. While the design and implementation of the harmonised tariff have been complex and have taken considerable time and effort across medical schools [1, 8], they clearly represent an excellent step towards better delivery of undergraduate medical education for students. Not only has the harmonised tariff delivered material shifts to support the delivery of changes in undergraduate medical education, but it has also begun to influence subtle yet important elements of the hidden curriculum. These changes may influence career intentions and better prepare graduates for the NHS’s shift [16] towards a more integrated, community-based model of care. Thus, by achieving funding parity between primary and secondary care, the tariff creates the necessary conditions for a curriculum that aligns more closely with the future needs of the health system and the patients it serves. As responsibilities transfer from NHSE to DHSC, recommendations for future practice based on our findings include:

1. Ensure information symmetry, transparent oversight and accountability. Continued use of Annex C and an external reporting tool will be crucial to maintaining consistency and transparency in operationalisation across medical schools.
2. Maintain formal budget-holder status. Ensure ongoing access to and control of primary care tariff budgets by those responsible for this teaching, as the designated budget holders, to support advocacy, agency and leverage within medical schools.
3. Align budget allocation with responsibilities and quality assurance. Support interrelationships between budget allocation and responsibilities to address elements of the hidden curriculum and support greater equity across undergraduate clinical education.

The findings may have relevance for secondary care, where centralised decision-making has reduced transparency and accountability for those delivering teaching [27, 28]. Secondary care could bring budget ownership closer to delivery by naming accountable leads at placement block or directorate level. Furthermore, secondary care could strengthen its governance by reinforcing the equivalent provisions in Annex B [34], which require NHS placement providers to demonstrate that tariff funding is used for placement delivery. 

Finally, while greater local oversight may support transparency, it is important not to lose the involvement of medical schools in primary care tariff governance. In contrast to secondary care, where funding now flows directly from NHS England to hospitals with little university input, the primary care model benefits from the combined educational and practical expertise of universities and teaching practices.

## Supplementary Information

Supplementary Material 1.

Supplementary Material 2.

## Data Availability

The datasets generated during the current study are not publicly available due to the risk of identifying participants given the small, specialist population of HUGPTs. Anonymised data summaries are available from the corresponding author upon reasonable request, subject to ethical and confidentiality considerations.
