# Supplementary material for: The impact of the harmonised education tariff on undergraduate general practice education in England: a qualitative interview study
Source: BMC Med Educ. 2026 Jun 17;26:1377. doi: 10.1186/s12909-026-09679-6 (PMC13508417; doi:10.1186/s12909-026-09679-6)
Supplement: Supplementary file 1 [file 12909_2026_9679_MOESM2_ESM.pdf]

Supplementary Materials

The Impact of the Harmonised Education Tariff on Undergraduate General Practice Education in England: A Qualitative Interview Study

Catharina Savelkoul and Sophie Park

Nuffield Department of Primary Care Health Sciences, University of Oxford

Contents of supplementary materials

Supplementary Table S1: Mapping of themes and subthemes to topic guide questions

Note on the Topic Guide

The core research questions remained fixed throughout the study; only the wording and sequencing of prompts were refined iteratively as preliminary analysis identified new areas warranting exploration (most notably, explicit questions about the NHSE reporting tool were added after early participants raised this topic). This iterative refinement of prompts within fixed research questions is consistent with best practice in reflexive thematic analysis [Braun & Clarke, 2021].

Question	Topic Guide Question Text
**TG Q1**	Could you start by telling me a bit about your role as HUGPT, how long you have been in post and the size and structure of your team?
**TG Q2**	Before we discuss the harmonised tariff in detail, could you briefly describe how undergraduate GP teaching was funded at your institution before March 2022?
**TG Q3**	How has the harmonised tariff changed the way funding for undergraduate GP education is managed at your institution, particularly in terms of who controls the budget?
**TG Q4**	How transparent is the budget allocation process now compared to before? Do you have full visibility of the funding coming in and how it is being spent?
**TG Q5**	Has the harmonised tariff enabled you to make any changes to the GP curriculum, for example, increasing the number of placement weeks or introducing new teaching activities?
**TG Q6**	Has the tariff affected your ability to recruit or expand your central GP teaching team or wider GP education workforce?
**TG Q7**	How has the tariff affected your ability to recruit and retain teaching practices? Have you noticed any changes in practice willingness to take students?
**TG Q8**	Has improved remuneration through the tariff changed the quality or quantity of teaching provided by GP tutors? If so, in what ways?
**TG Q9**	Have there been any changes to quality assurance processes for GP placements as a result of the tariff?
**TG Q10**	From what you have observed, how has the tariff affected the student experience of GP placements, for example, in terms of placement logistics, tutor engagement, or student attitudes towards general practice?
**TG Q11**	How useful have you found the Annex C guidance document and the NHSE reporting tool? How do you use them in practice?
**TG Q12**	Have there been any challenges or negative consequences of the tariff, for example, increased administration, difficulties with Annex C interpretation, or concerns about sustainability?
**TG Q13**	Looking ahead, given the planned abolition of NHSE and the Government's 10-Year Health Plan, what do you see as the key priorities for the future of undergraduate GP education funding?

Supplementary Table S1: Mapping of Themes and Subthemes to Topic Guide Questions

Theme	Subtheme	n participants contributing	Topic Guide Question(s)	Topic Guide Question Text (inferred from research questions)	How Theme Emerged
**1. Budget control, transparency and organisational change**	**1a. Greater transparency and oversight**	n=11/12 participants (n=10/10 English)	**TG Q3, TG Q4**	*Q3: How has the harmonised tariff changed budget management? Q4: How transparent is the budget allocation process now compared to before?*	**Direct question**<br>Emerged consistently as the most prominent finding in response to direct questions about budget management and oversight. All but one participant described improved visibility of funding flows.
	**1b. Gaining formal control of the budget**	n=9/10 English participants	**TG Q3, TG Q4**	*Q3: Who controls the budget under the harmonised tariff? Q4: Has formal designation as budget holder changed your position within the medical school?*	**Direct question**<br>Emerged directly from questions about budget management; participants described the shift in institutional power dynamics. The 'sign off' function and ability to argue for resources arose prominently.
	**1c. Institutional cultural change**	n=5/12 participants	**TG Q2, TG Q3**	*Prompted by broader discussion of what funding parity means symbolically, beyond financial mechanics.*	**Participant-initiated (extended from direct questions on budgeting)**<br>Several participants spontaneously introduced the symbolic significance of funding parity (its effect on GP's perceived prestige within medical schools) when discussing the broader implication of the tariff. This was not an explicit question in the initial topic guide but was retained as a prompt after it arose in early interviews.
**2. Curriculum expansion and educational innovation**	**2a. Expansion of GP teaching within the curriculum**	n=7/10 English participants	**TG Q5**	*Q5: Has the harmonised tariff enabled you to make any changes to the GP curriculum — for example, increasing the number of placement weeks or introducing new teaching activities?*	**Direct question**<br>Emerged directly from the curriculum question. Participants described using HUGPT budget control to increase the proportion of curriculum time in general practice.
	**2b. Central GP teaching team (CGPT) growth**	n=6/10 English participants	**TG Q6**	*Q6: Has the tariff affected your ability to recruit or expand your central GP teaching team or wider GP education workforce?*	**Direct question**<br>Arose directly from questions about team resourcing. Participants described moving from under-resourced, ad hoc arrangements to structured teams.
	**2c. Development of educational initiatives**	n=5/10 English participants	**TG Q5, TG Q6**	*Q5 and Q6 combined; participants described new creative initiatives that had become possible with ring-fenced funding.*	**Direct question (extended response)**<br>Participants extended their responses to curriculum and team questions to describe specific innovative projects enabled by the new funding model (e.g., clinical humanities projects, simulated clinics).

Theme	Subtheme	n participants contributing	Topic Guide Question(s)	Topic Guide Question Text (inferred from research questions)	How Theme Emerged
**3. Placement capacity**	**3a. Recruitment of new teaching practices**	n=9/10 English participants	**TG Q7**	*Q7: How has the tariff affected your ability to recruit and retain teaching practices? Have you noticed any changes in practice willingness to take students?*	**Direct question**<br>One of the most consistently reported findings; participants described practices previously unwilling to teach now agreeing to participate following improved remuneration.
	**3b. Retention of existing teaching practices**	n=8/10 English participants	**TG Q7, TG Q8**	*Q7 (retention element): Have existing practices changed their behaviour regarding student hosting since the tariff? Q8: Has remuneration affected teaching stability?*	**Direct question**<br>Directly prompted by the retention component of the placement recruitment question. Participants described practices that had threatened to stop teaching, with the tariff reversing this trend.
	**3c. Ongoing concerns over placement capacity**	n=7/10 English participants	**TG Q12, TG Q13**	*Q12: Are there any ongoing challenges? Q13: Looking ahead, what are the key risks for undergraduate GP education?*	**Participant-initiated (also prompted by challenges/future questions)**<br>While touched on by the challenges and future-outlook questions, the depth of concern about medical school expansion, new schools, and competition for practices was largely participant-driven. This subtheme was formalised as a prompt after it arose prominently in early interviews.
**4. Improving authentic teaching quality and assurance**	**4a. Tutor engagement**	n=6/10 English participants	**TG Q8**	*Q8: Has improved remuneration through the tariff changed the quality or quantity of teaching provided by GP tutors? If so, in what ways?*	**Direct question**<br>Arose directly from the tutor engagement/quality question. Participants described a perceived (though not formally measured) improvement in tutor motivation and quality.
	**4b. Quality assurance processes**	n=5/10 English participants	**TG Q9, TG Q11**	*Q9: Have there been any changes to quality assurance processes for GP placements as a result of the tariff? Q11: How do you use Annex C guidance in QA conversations?*	**Direct question (with extension from Annex C question)**<br>Emerged from the quality assurance question; participants described how having formal budget authority allowed them to leverage funding as a quality mechanism. Some described the Annex C document as a tool to hold practices accountable.
**5. Impact on student experience**	**5a. Student perceptions of general practice**	n=4/10 English participants	**TG Q10**	*Q10: From what you have observed, how has the tariff affected the student experience of GP placements — in terms of student attitudes towards general practice?*	**Direct question**<br>Arose from the student experience question. Participants were cautious about overclaiming a direct link to career intentions, noting many confounding factors.
	**5b. Practical benefits for students (logistics and placement quality)**	n=5/10 English participants (logistics); n=3/10 (acting on feedback)	**TG Q10**	*Q10: How has the tariff affected the student experience of GP placements — for example, in terms of placement logistics, placement length, or tutor engagement?*	**Direct question (extended response)**<br>Participants extended their student experience responses to describe concrete improvements in placement logistics (e.g., practices agreeing to teach closer to students' homes) and how tariff funding enabled schools to act on student feedback (e.g., funding full-day placements).

Theme	Subtheme	n participants contributing	Topic Guide Question(s)	Topic Guide Question Text (inferred from research questions)	How Theme Emerged
**6. Accountability frameworks**	**6a. Annex C as guidance and legitimacy tool**	n=8/10 English participants	**TG Q11**	*Q11: How useful have you found the Annex C guidance document and the NHSE reporting tool? How do you use them in practice?*	**Direct question**<br>Arose directly from the accountability question. Participants described Annex C serving a dual function: providing internal clarity on tariff use and acting as a legitimacy tool in negotiations with medical school finance colleagues.
	**6b. Use of the NHSE reporting tool**	n=7/10 English participants support for mandatory reporting; n=6/10 English emphasised importance	**TG Q11**	*Q11: Have you used the NHSE reporting tool? What do you think about the requirement to report on tariff use? Should this continue under the new DHSC arrangements?*	**Direct question; reporting tool added as explicit prompt after early participants raised it spontaneously**<br>The reporting tool question was not in the initial topic guide but was added as an explicit prompt after it arose in early interviews. Consistent with good practice in reflexive thematic analysis, the core research questions remained unchanged while this emergent area was formalised as a prompt.
**7. Challenges and limitations of the tariff**	**7a. Administrative burden of the reporting tool**	n=4/10 English participants	**TG Q12**	*Q12: Have there been any challenges or negative consequences of the tariff — for example, increased administration?*	**Direct question**<br>Arose from the challenges question. Participants described the reporting tool as valuable in principle but time-consuming in practice.
	**7b. Difficulties interpreting Annex C in edge cases**	n=2/10 English participants	**TG Q11, TG Q12**	*Q11/12: Have you experienced any difficulties applying Annex C guidance — for example, in ambiguous situations about what counts as eligible expenditure?*	**Participant-initiated (extended from Annex C discussion)**<br>Arose spontaneously during discussions about Annex C as a tool. Two participants described specific situations where the guidance did not clearly resolve their questions about eligible expenditure.

Note on participant counts: All participant counts are reported using the 10 English participants as the denominator.

Abbreviations: TG = Topic Guide; HUGPT = Head of Undergraduate GP Teaching; NHSE = NHS England; DHSC = Department of Health and Social Care; Annex C = Annex C of the 2022–23 Education and Training Tariff guidance document, setting out the scope of undergraduate primary care placements funding.
